# A Case of Spontaneous Atraumatic Renal Bleeding (Wunderlich Syndrome)

**DOI:** 10.7759/cureus.93311

**Published:** 2025-09-26

**Authors:** Lydia MY Chang, Shail Shah, Dora Moon

**Affiliations:** 1 Core Surgical Training, Health Education England North West Deanery, Northwest, GBR; 2 Urology, Lancashire Teaching Hospitals National Health Service (NHS) Trust, Preston, GBR; 3 Urology Specialty Training, Health Education England North West Deanery, Northwest, GBR

**Keywords:** atraumatic, pseudoaneurysm, pseudoaneurysm coiling and embolization, renal bleed, urology emergency

## Abstract

We present an uncommon case of atraumatic spontaneous renal hemorrhage in a 72-year-old woman with a background of atrial fibrillation on edoxaban who presented with a sudden onset of left abdominal pain, nausea, vomiting, lethargy, and dizziness. Cross-sectional imaging was performed and confirmed an ongoing renal bleed. She had a trial of conservative management; however, repeated imaging showed an ongoing, slow but active renal arterial bleed; hence, she underwent an embolization procedure. On-table angiogram revealed pseudoaneurysms in the upper and lower poles of the kidney, for which she underwent successful renal artery embolization.

## Introduction

Atraumatic spontaneous renal hemorrhage, also known as Wunderlich syndrome, is a potentially life-threatening urological emergency. It is eponymously named after German physician Carl Wunderlich, who first described a case in the year 1856 of a patient with spontaneous renal hemorrhage in the subcapsular and perirenal space in the absence of trauma. Patients may present with Lenk's triad of acute flank pain, palpable flank mass, and hypovolemia, though this may not always be the case [1]. It is a rare occurrence, with only approximately 450 published cases between 1933 and 2000 [2]. The etiology can be varied but is associated with underlying renal neoplasms, vascular malformations or disorders, renal cysts, and coagulation disorders or anticoagulant therapy [3]. Though direct oral anticoagulants (DOACs) have significantly lower risks compared to warfarin, it has been reported that there is up to 4% risk of major bleeding [4]. Early recognition is crucial to prevent mortality. Management options include both surgical and conservative management, depending on the patient's condition [5].

## Case presentation

**Figure 1:**
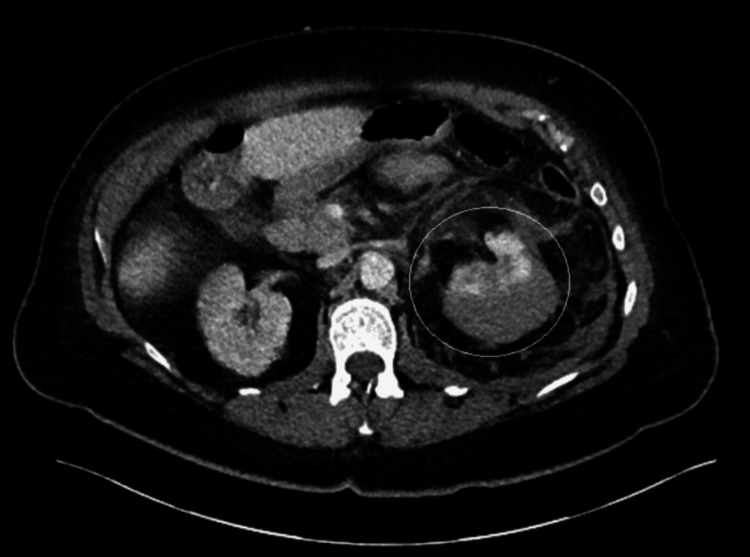
CT of the abdomen pelvis showing acute left perinephric/subcapsular hematoma CT: computed tomography

**Figure 2:**
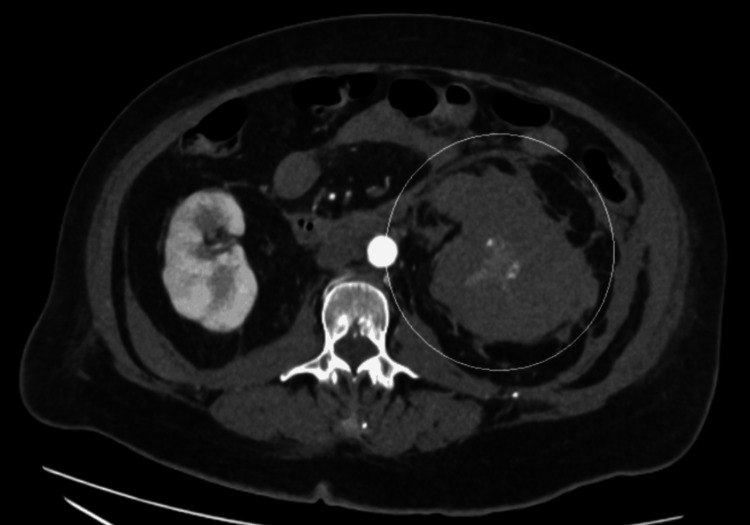
CT angiogram showing increase in the size of left perinephric/subcapsular hematoma

**Figure 3:**
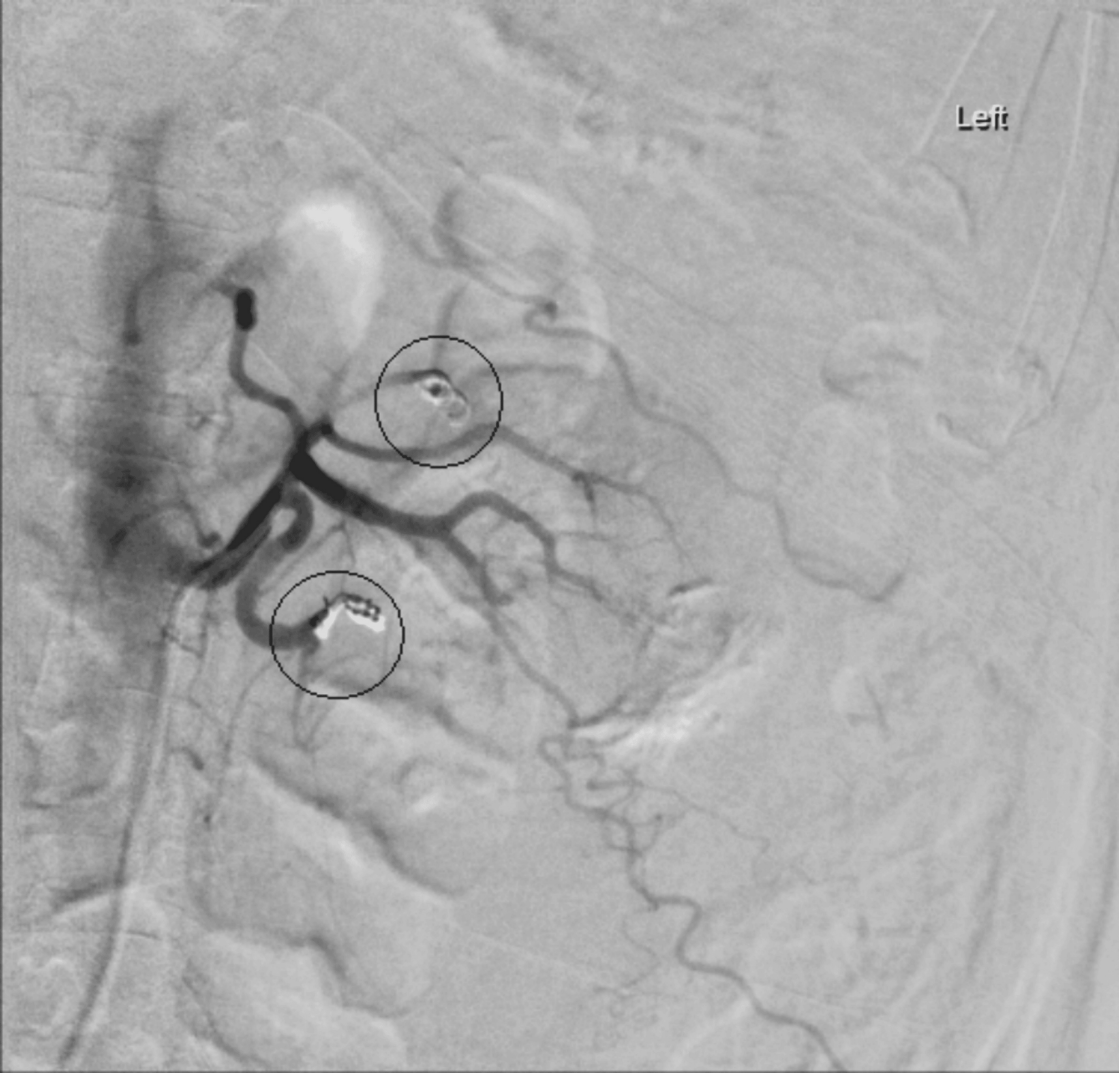
Embolization of pseudoaneurysms in the lower and upper poles of the left kidney with microcoils

A 72-year-old woman presented to the emergency department of a district general hospital with complaints of sudden onset of left-sided abdominal pain, nausea, vomiting, generalized weakness, and dizziness. She had a past medical history of atrial fibrillation, anticoagulated with edoxaban, gout, hypertension, and chronic kidney disease stage 3. Upon examination, she was found to be tender in the left iliac fossa, with no visible bruising. Initial computed tomography (CT) of the abdomen and pelvis reported an acute left perinephric/subcapsular hematoma (Figure 1). She was started on prothrombin complex concentrate. Despite this, serial hemoglobin monitoring showed a decreasing trend (Table 1) from a baseline of 177 to 94 g/L in the span of 24 hours; therefore, a CT angiogram was performed the following day, which revealed an increase in the size of the perinephric hematoma with small focal areas of contrast extravasation suggestive of slow active arterial bleeding (Figure 2). She was then referred to our tertiary center for embolization. Two pseudoaneurysms were found during on-table angiography: one in the lower pole of the kidney and another in the segmental upper pole artery. Both pseudoaneurysms were embolized and occluded with microcoils successfully (Figure 3). Her renal function improved to near baseline three days after the procedure. She was discharged after a few days of hemodynamic stability and stable hemoglobin levels after the procedure. Her anticoagulant was continued to be withheld for two weeks after the procedure.

**Table 1:** Laboratory investigations showing decreasing trend of hemoglobin

Day of admission	Hemoglobin levels, g/L (reference range: 115-165 g/L)
1	177
1	159
1	149
2	109
2	94

## Discussion

Spontaneous atraumatic renal hemorrhage has various etiologies. Renal neoplasms (angiomyolipomas and renal cell carcinomas) were the most common cause, followed by vasculitides such as polyarteritis nodosa [2]. Angiomyolipomas, specifically those larger than 4 cm, remain the most common cause. Infection was reported as the cause in a small number of patients [6]. In some cases, no cause was identified despite a thorough work-up [3].

The diagnosis of spontaneous atraumatic renal hemorrhage may be challenging, as it can present similarly to an acute abdomen, such as aortic dissection, ruptured abdominal aneurysm, or perforated viscus. While ultrasound of the kidney is rapid and noninvasive, it often does not contribute to the diagnosis, thus favoring CT scan as the diagnostic imaging modality of choice [7].

Management of spontaneous atraumatic renal hemorrhage includes initial resuscitation and stabilization, followed by either conservative management with close observation, arterial embolization, or surgery, depending on the hemodynamic status and findings on imaging (whether there is the presence of active bleeding). Management has shifted over the years with the increasing availability of interventional radiology in an increasing number of centers. Patients with active bleeding previously underwent emergency nephrectomies as first-line treatment; however, there has been a decreasing trend in surgical management, and selective arterial embolization has now gained a pertinent role in the management of spontaneous atraumatic renal hemorrhage, with many recommending it as a definitive treatment [2]. Selective arterial embolization is a low-risk and minimally invasive procedure compared to nephrectomy, while also significantly improving renal function compared to conservative management and being organ-preserving [8].

## Conclusions

Atraumatic spontaneous renal hemorrhage (Wunderlich syndrome) is a rare but potentially life-threatening urological emergency. The most common causes are renal neoplasms such as angiomyolipoma and renal cell carcinoma; however, idiopathic cases do occur. It is also important to remember that DOACs, while having a much lower risk of bleeding compared to older agents like warfarin, still remain a risk factor for major bleeding. Contrast-enhanced CT is essential for diagnosing and assessing the underlying cause and extent of bleeding. Management strategies range from conservative monitoring to interventional radiological embolization or surgical intervention, depending on hemodynamic stability and underlying pathology.
